# Conducting In-Depth Interviews via Mobile Phone with Persons with Common Mental Disorders and Multimorbidity: The Challenges and Advantages as Experienced by Participants and Researchers

**DOI:** 10.3390/ijerph182211828

**Published:** 2021-11-11

**Authors:** Azadé Azad, Elisabet Sernbo, Veronica Svärd, Lisa Holmlund, Elisabeth Björk Brämberg

**Affiliations:** 1 Department of Psychology, Stockholm University, SE-106 91 Stockholm, Sweden; azade.azad@psychology.su.se; 2 Department of Social Work, University of Gothenburg, SE-405 30 Gothenburg, Sweden; elisabet.sernbo@socwork.gu.se; 3 Department of Social Work, Södertörn University, SE-141 89 Huddinge, Sweden; 4 Division of Insurance Medicine, Department of Clinical Neuroscience, Karolinska Institutet, SE-171 77 Stockholm, Sweden; 5 Unit of Intervention and Implementation Research for Worker Health, Institute of Environmental Medicine, Karolinska Institutet, SE-171 77 Stockholm, Sweden; lisa.holmlund@ki.se (L.H.); elisabeth.bjork.bramberg@ki.se (E.B.B.)

**Keywords:** data collection, telephone interview, semi-structured interview, COVID-19 pandemic, common mental disorders, multimorbidity, emotion work

## Abstract

Qualitative interviews are generally conducted in person. As the coronavirus pandemic (COVID-19) prevents in-person interviews, methodological studies which investigate the use of the telephone for persons with different illness experiences are needed. The aim was to explore experiences of the use of telephone during semi-structured research interviews, from the perspective of participants and researchers. Data were collected from mobile phone interviews with 32 individuals who had common mental disorders or multimorbidity which were analyzed thematically, as well as field notes reflecting researchers’ experiences. The findings reveal several advantages of conducting interviews using mobile phones: flexibility, balanced anonymity and power relations, as well as a positive effect on self-disclosure and emotional display (leading to less emotional work and social responsibility). Challenges included the loss of human encounter, intense listening, and worries about technology, as well as sounds or disturbances in the environment. However, the positive aspects of not seeing each other were regarded as more important. In addition, we present some strategies before, during, and after conducting telephone interviews. Telephone interviews can be a valuable first option for data collection, allowing more individuals to be given a fair opportunity to share their experiences.

## 1. Introduction

In-depth interviews are one of the most common forms of data gathering in qualitative research [1,2]. The purpose is to obtain information about how individuals view, understand, and make sense of their lives, and how they assign meaning to particular experiences, events, and subjects [3]. Hence, such interviews are appropriate for exploring phenomena about which we have limited knowledge, or in generating knowledge to inform social or healthcare interventions [4,5,6,7,8].

Qualitative interviews have traditionally been conducted in-person, either individually or in focus groups [3,5]. There seems to be a consensus in the literature that in-person interviews are the best (‘gold standard’) format [9]. However, they are not always possible due to logistical, practical, or safety reasons, such as the COVID-19 pandemic [10,11,12]. The COVID-19 pandemic has produced a wide range of changes in customary practices of conducting research, particularly on the gathering of data [13]. Researchers, ourselves included, have been forced to use remote methods, such as telephone interviews as a mean of collecting qualitative data. Although proven to be a viable way of data collection [14], there is still a lack of methodological discussion about the use of telephone interviews for certain groups of participants [15], such as persons with common mental disorders (CMDs) (i.e., depression, anxiety, adjustment disorders) or multimorbidity. These groups, with symptoms of e.g., exhaustion and bodily aches, have been difficult to recruit to research studies, due to mental distress, medications, stigma, and a reduced capacity to take on new information and thus to consent to participation, for example [16,17]. Telephone interviews might be a well-suited solution for these groups [18]; however, there are a lack of studies investigating the experiences of telephone interviews from the perspective of people with CMDs and multimorbidity.

### Telephone Interview as a Method of Collecting Qualitative Data

Previously, telephone interviews have been used as a last resort for collecting qualitative research data [3,19,20]. The most common concerns about telephone interviews are that they might have a negative impact on the richness and quality of the collected information [19], the challenges in establishing rapport [21,22], and the inability to respond to visual and emotional cues [15]. Other criticisms involve the increased risk of misunderstandings and the inability to know if and when to ask probing questions or introduce more sensitive topics [20]. However, a growing body of literature using the telephone as a way of collecting data, as well as studies comparing the use of telephone with in-person interviews, do not find support for the traditionalist view. Rather, scholars make the case for the potential of in-depth telephone interviews as a viable and equivalent option for qualitative research [23], with some even arguing that they are, in some regards, methodologically superior to in-person interviews [24,25].

Available studies have, for example, shown that telephone interviews generate the same amount of data richness as in-person interviews in terms of word count and topic-related information [26,27], and only modest differences in depth of data [28], even though telephone interviews tend to be shorter [29]. One study [14] found that in-person interviews are more conversational and detailed than remote methods (telephone and Skype), but that they do not clearly lead to differences in interview ratings. Other scholars [30] state that telephone offer flexibility regarding when and where to conduct the interview [24], which increase anonymity and reduce distraction (for interviewees), thus improving the information given [26,31]. Several attempts to develop tools improving the success of in-depth telephone interviewing have been made [32,33,34,35], considering the criticisms raised against telephone interviews, as well as the counter arguments. These tools provide a set of comprehensive approaches to follow before, during, and after the interview to ensure effective use. These emphasize the significance of communicating the importance of participant contribution, explaining the purpose of the study in the early phase of the research either in writing or initial telephone contact, and establishing rapport through small talk when first contacting the participant [32]. Because of the absence of non-verbal cues and difficulties in identifying visual emotional expressions, the importance of providing verbal feedback and follow up probes are stressed [36], as well as using vocalizations and clarification to show responsiveness [32]. Such verbal cues or probed questions can in turn result in both parties listening more carefully [30].

Studies investigating the use of telephone interviews from the perspective of the interviewee have mostly yielded positive results. For many, telephone interviews are the preferred choice, when given the option to choose [25], for reasons of convenience and greater anonymity [35,37]. In contrast to traditionalist views, some researchers have found that interviewees find it is easy to establish rapport [23]. Hence, some authors claim that telephone interviewing is suitable for vulnerable and marginalized populations and more sensitive questions [32,35].

Telephone interviews can also have advantages for the interviewer, by reducing self-consciousness [24] and bias and stereotyping about the interviewer. It can also benefit the researcher–participant relationship by providing a more balanced power dynamic between the two [27]. 

One group of participants who, despite the growing body of literature examining the advantages and challenges of telephone interviews, have not been further investigated, are people with experience of sick leave due to illness, such as CMDs and/or multimorbidity. It has been argued that there are specific challenges in interviewing people with mental illnesses and barriers having to do with the consequences of their symptoms (such as mental distress, medications, stigma, reduced ability to take in new information, and passive interaction with healthcare professionals) [16,17,38]. Research has also shown that recent illness or present ill health affect research participation negatively, and using telephone interviews has been suggested as a way of enhancing response rate [18]. Including the experiences of people who are or have been on sick leave due to CMDs or multimorbidity in research is critical, due to, for example, the individual and societal burden. However, in doing so, the interview situation must be adapted to suit the participants needs. This may be provided by conducting telephone interviews.

The aim of the present study is, therefore, to explore the use of the telephone for semi-structured interviews from the perspective of these individuals. A further aim is to address the challenges and advantages of using the telephone from the perspective of the interviewer. To the best of our knowledge, there are no previous methodological studies into the use of telephone interviews with individuals with CMD or multimorbidity. Our study is, therefore, a unique contribution to the scarce research available on this topic.

## 2. Materials and Methods

### 2.1. Study Design

This study used a qualitative approach involving semi-structured interviews with people with CMD or multimorbidity with on-going sick leave, or who had returned to work after sick leave. The interviews reflect the participants’ unique experiences regarding the use of mobile phone when collecting data. The participants are included in two different projects (see Table 1). In these projects, in-person interviews were changed to telephone interviews because of the COVID-19 pandemic. This study focuses on the last part of the interviews where probes were added to take account the participants experience of being interviewed by mobile phone. We primarily refer to mobile phones, as ownership of mobile phone is generally, and in Sweden in particular, much higher than landline ownership [39]. Both participants and researchers used mobile phones during the interviews.

### 2.2. Participants

Participants were recruited from two projects: the RECO-project [40,41] and the PROSA-project [42] (see Table 1). All participants were given written and/or oral information by post about the study, including that participation was voluntary. In the RECO-project, 70 individuals received written information, of whom 13 replied that they were interested in participating. One person later declined to participate because their knowledge of the investigated subject in the particular project was limited. In one of the PROSA-projects, 49 individuals were given oral information about the study. Of those, 18 received written information and agreed to be contacted by the researcher. Of these, 10 took part in an interview. In the other study linked to the PROSA-project, 15 participants were contacted by telephone by the researcher for information. Of these, three did not answer, one did not fit eligibility criteria, one declined to participate, and 10 were included in the present study. 

In total, 32 participants were included in this study. Twelve participants were on sick leave due to multimorbidity, and twenty were on sick leave or had recently returned to work after sick leave due to CMDs. The participants represent a variety in ages (ages ranged from 22 to 62) and gender, although a majority were women (7 men and 25 women) and type of employment. For more detailed information about the participants, see Table 2.

### 2.3. Data Collection

Data were gathered through semi-structured mobile phone interviews with the participants and field notes kept by the researchers. The interviews were conducted between March and September 2020. The interviews followed interview guides with primary questions specifically for each project, and follow-up probes about being interviewed by telephone. Only the data relating to telephone interviewing are included in the present study. The probes addressed the participants’ experience of the conducted telephone interviews, including the challenges and advantages of being interviewed over the telephone. The participants were also asked to reflect over possible alternative modes of interviews (such as in-person or internet-based methods). Their reflections are not to be understood as direct comparisons between the use of different research methodologies, as they only partook in telephone interviews and not internet-based, or in-person interview methods. Rather, the participants experiences are to be understood as unique reflections on being interviewed using mobile phones. During the interviews, the participants reflect on experiences of meeting professionals in-person and/or working with different technologies.

Interviews ranged in length from about 30 to 90 min which included the whole interview. Three members of the research team (first, second and fourth author) conducted the interviews. All members of the research team were experienced in conducting in-depth in-person interviews, and some had also previous experience of conducting telephone interviews. Interviews were digitally recorded and transcribed verbatim in Swedish. The transcripts and digital recordings were cross-checked.

The data also consist of field notes [43] with reflections upon our experience as researchers conducting in-depth in-person and telephone interviews as a means of data collection. The field notes were written down directly after every phone call. Each interviewer noted their immediate recollection of the conversation, summarizing how they experienced the interview format and content as well as their reflections about the interview generally. 

### 2.4. Data Analysis

Thematic analysis [44,45] was conducted to explore participants’ views of participating in qualitative interviews by telephone. We began our analysis by reading through the transcribed text to familiarize ourselves with the material and search for patterns in the data. We then identified important and interesting features focusing on the semantic and latent meanings in line with the aim. These features included words, sentences, or paragraphs relating to what the participants found difficult or easy with being interviewed over telephone, and were then condensed and assigned a code. The third step involved searching for possible themes, by identifying and coding them across participants. This step was performed on the first 22 interviews collected and refocused the analysis at the broader level of themes, rather than codes, and involved sorting the codes into potential themes and collating all the relevant coded data extracts within these themes. The first and second author made a first draft of the themes and the remaining researchers read through and discussed them. This discussion involved reviewing and refining themes, both with regard to each theme in itself and in relation to the data set. The ten remaining transcripts were analyzed based on the drafted themes and used to check for depth in the analysis. No new themes were added and the initial themes were adjusted until the conceptual depth in the themes was agreed upon [46]. A final step involved rechecking the data to code additional codes that may have been missed, before refining and defining the essence of each theme by naming them. During the analysis process, the coding and themes were repeatedly discussed by all the researchers until consensus was reached. During the analysis process, the first author translated the themes and quotes from Swedish into English and the second and the fourth authors reviewed the translations, before all the authors made a final revision.

The field notes are understood as condensed rather than transcribed, and were jointly discussed and elaborated, inspired by notions on how the written record and memory interact [47]. Our reflections based on these field notes are analyzed and presented separately from the analysis of the participants’ narratives. This analysis was inspired by thematic analysis, although not following Braun and Clarke’s [44,45] six steps.

## 3. Results and Discussion

The findings are presented in three themes, including discussion in relation to relevant research: flexibility of location, personal well-being and emotional ease, and balancing anonymity and social responsibility. The themes reflect patterns of meaning relating to the experiences of being interviewed over the mobile phone. They are not hierarchical in relation to one another but rather presuppose each other; one enables the other while being on the same analytical level. After presenting the three themes, the researchers’ experiences and reflections are offered and discussed in relation to the themes.

### 3.1. Flexibility of Location

The first theme had to do with practical and environmental aspects, such as the flexibility to choose place and surrounding during the mobile phone interview, compared to landline phone or in-person options. The flexibility of using mobile phones meant that the participants were free to choose place for the interview, and did not have to physically meet the interviewer. Most participants conducted the interviews from home, and a few from their workplace—geographically close and familiar environments. Not having to spend time or energy travelling was of great importance for the majority of the participants. The time saved in telephone interview compared to in-person was, for some participants, crucial for participation. For example, one participant said:

It’s also nice to be at home and not have to go to an interview and so on, because that would use so much energy. Then maybe I would choose not to do it. (Female, 38 years, multimorbidity)

Although these benefits—for both participants and researchers—have been identified in previous research [24,26], our results point to the importance of flexibility, both regarding geography and time for this group of participants specifically. As their mental and/or physical health makes it difficult for them to travel, telephone interviews offer a way of participating without having to do so.

Flexibility was also associated with the specificity of the mobile phone rather than other choices of technology, for example internet-based voice options, such as Skype or Zoom. While some thought that internet-based video options were desirable because of the ability to see each other, the vast majority preferred the mobile phone option. As one participant said:

I would also have worried about the [internet-based] technology, I have to say, it’s probably inevitable that you do to some degree. (Female, 34 years, stress syndrome)

Using the mobile phone, however, added no extra technical demands for the participant and, therefore, meant limited technical worries before and/or during the interview. Some used internet-based technology at work, but others had no experience of such tools and said they would have been worried about coping with the technology. This is in line with Seitz’s [48] reasoning that technical difficulties may have a negative impact on the interview. For our participants, in contrast to what other researchers have purposed, Sipes et al.’s [49] voice-only options are not always the equal option to using mobile phones. 

### 3.2. Personal Well-Being and Emotional Ease

In personal and emotional terms, using mobile phone rather than in-person interviews was seen as helping the participants’ well-being and emotional ease. Suffering from CMD and/or multimorbidity was already perceived as demanding by the participants. In comparison to an in-person interview or internet-video based options, the mobile phone interview not only enabled them to choose place and surrounding for the interview, but also position and the ability to move around while talking. Some participants appreciated the ability to conduct the interview via mobile phone while having a walk outside, which had not been possible using landline phone. Being physically comfortable and free was highly valued, given that the participants had symptoms of CMDs and/or multimorbidity with depression, exhaustion, and bodily aches. In line with Cachia and Millward’s findings [24], our participants reported being less self-conscious while not having to think about how to sit or conform to social cues and norms as in an in-person or video-based meeting. 

Being able to do the interview over the telephone caused less anxiety and was less emotionally demanding. This is described by one of the participants:

There’s a lot of fear and stress, and talking about these things can make it, since it’s so personal, I get scared of being judged and looking someone in the eye, seeing them react in a negative way about something that has… You can’t see that on the phone. (Female, 50 years, multimorbidity)

Other emotional advantages had to do with feeling less inhibited when not being able to see each other. For some, this meant being able to talk more freely; for others, it meant displaying more emotions such as crying. For example, one participant said that it was easier to continue talking even though she had been crying, because the interviewer may not even have noticed. The telephone was experienced as providing a positive sense of protection when sharing. As one participant put it:

When you get an anxiety attack, or, I don’t know how to put it, but like, you feel kind of protected behind the phone. (Male, 25 years, depression)

In this regard, conducting the interviews over the telephone led to fewer emotions being visible, so it was easier to cry than when meeting someone in person. For some participants, the less emotion work demanded by telephone interviews was a precondition for participation. These findings reinforce those of previous studies [37,50], showing that some participants regarded the telephone interview as the ‘only option’ for them being able to participate at all. This suggests that telephone interviews can increase participation and, thus, the heterogeneity and breadth of the data. In particular, it seems to be crucial for being able to involve some of the most vulnerable groups, i.e., those with limited energy and an ability to participate in an in-person interview due to mental or physical illness. As such groups have been outlined as hard to recruit for research studies [16], our results point to that telephone interviews might help overcome the challenges in interviewing people with for example CMDs and/or multimorbidity. Using the telephone can simply be considered as an easier way to participate in research interviews, by placing less demands on the participant compared to video options or face-to-face interviews.

These findings also relate to how telephone interviews reduce participants’ emotion work in accordance to Hochschild [51], because they do not visibly convey and manage their feelings in the social interaction. Goffman [52] argues that people strive to convey their feelings in a socially acceptable way and manage their emotional expressions and impressions. By removing the visible dimensions of social interaction, and giving participants the opportunity to be ‘protected behind the phone’, the emotion work is not completely removed from the interaction, but the conditions are changed because participants can maintain the desired anonymity and emotional distance. The telephone interview, compared with in-person interviews, allows interviewees to shed an unseen tear, lie down without anybody knowing, and keep visible emotions private. The freedom offered by these choices, together with the flexibility and time- and energy-saving aspects discussed earlier, suggest that telephone interviews allow participants to share their experiences while putting less strain on them as they do so.

### 3.3. Balancing Anonymity and Social Responsibility

The third theme focuses more explicitly on the relational aspects of the mobile phone interview. The physical distance, with the participant and interviewer unable to see each other, did not only make it easier to protect your emotional expressions, but also created a sense of anonymity, making it easier to talk about sensitive subjects. As one of the participants put it:

It gets very personal, these are very personal things to talk about … and I don’t know you. So then it can be nice to have this little bit of distance. (Male, 46 years, depression)

The sense of freedom related to the ability to choose the level of intimacy in the interview, unique to the telephone mode, thus contributing to a sense of anonymity and psychological distance. This also made it more likely for interviewees to feel comfortable talking about sensitive subjects [25,37]. The perceived higher degree of anonymity might result in richer data and a higher validity among responses, as the telephone mode could decrease social desirability. For example, avoiding being seen by an in-person or video-based interviewer can create a feeling of being less judged and not being in the gaze of the professional [25]. Telephone interviews can thus lead to a more balanced power dynamic between the participant and the interviewer [27]. The feeling of distance was also described as making it easier to take control and end a conversation which may not have felt good or right.

Telephone interviews required less social responsibility since participants were able to focus solely on what the other person was saying instead of thinking about social cues and norms as in an in-person meeting (such as where to look, how to sit, when to nod or smile, and so on). Goffman [52] uses the term impression management to discuss how people put on performances during in-person social interactions in order to manage, rather than show, their feelings. Our findings suggest that the telephone interview may ease the burden on the participants to put on a performance, as they do not have to think about their body language, or relate to social clues or norms to the same extent as in an in-person interview. 

The downside of this form of interview was the required intense listening, which is described as somewhat demanding by some participants. Receiving fewer cues via visual interaction is, thus, described as a balancing act, as some participants stressed the importance of the interviewer keeping the conversation on track, not leaving them unsure about whether or not they are talking about the ‘right’ things. They mentioned the importance of the interviewer’s voice, both in relation to being able to understand the other and being understood. For example, participants described finding it easy to ‘get a feeling’ for the other person through the tone of voice instead of through the other social cues used when you are sitting face to face. As one of the participants explained, the way the interviewer spoke, referring to the tone of voice, helped to install confidence. Although verbalization has been stressed in telephone interviews [32], our finding adds to research by stressing the importance of not only what is being said but also how it is said. The importance of tone and attribute of the interviewers’ voice is, thus, a crucial tool to use within in-depth telephone interviewing. 

When talking about the negative aspects of telephone interviews, the participants also mentioned several factors in the first contact and impression of an in-person meeting. For example, they mentioned that it is interesting and fun to meet new people and that it is nice to see the other person. This was often linked to curiosity and ‘the human encounter’. Negative aspects of not being able to see each other were also described to affect interactions:

Not that I find it difficult, but if you’re sitting together, in a way you have another kind of interplay because you can see one and other. (Female, 46 years, multimorbidity)

However, because they viewed this interview as a one-off and were not going to have a further relationship with the person interviewing them, the positive aspects of not seeing each other were regarded as more important. As they explained, they were first and foremost interested in conveying their experiences. Some also reported that they were able to create their own image of the interviewer, which filled the same function as an in-person meeting.

### 3.4. Researchers’ Experiences and Reflections

The analysis of the researchers’ experiences and field notes resulted in two themes having to do with *worries and challenges about the technology* and *relational and social aspects*, as well as a third overarching theme of understanding *the telephone as a ‘shield’*. Quotations from our field notes are provided for each theme in order to illustrate and contextualize the results. Regarding the first theme, *worries and challenges about the technology*, the researchers reflected on that the mobile phone interview was sometimes imbued with worries and challenges about the technology used, for example not being able to control the quality of participants’ network coverage or mobile equipment. Using mobile phone can, therefore, involve more challenges regarding technology compared to using landline phone. Moreover, the participants´ choice of environment in some cases meant disturbances that challenged the researchers´ sense of being able to control the interview. The possible negative impact on the interview if, for example, the interviewees network coverage was insufficient, or if there were disturbances in the physical or social environment is illustrated by this reflection:

The first time I call him, he is in his car, and we agree that I can call again in 15 min, when he has arrived home. At the beginning of the interview, it is somewhat difficult because he has not found a friend for his son to play with [as he had hoped] and he is a bit hesitant related to what he can do to occupy his son. I offer to reschedule, but he wants to do the interview and starts a movie [that his son can watch during the interview]. (Written by L.H. The quote refers to a male participant, 45 years, stress syndrome)

The participants being in a situation where they can decide if they want to wash their dishes or take a stroll while talking in their mobile phone can leave the researcher experiencing loss of power over the situation. This disadvantage for the researcher can be an advantage for the participant, showing that using the telephone for interviewing involves giving away some power over the situation to the interviewee. Whale [50] points to the loss of power for the researcher, in interviewing over Skype or telephone, as something that enables a more balanced power dynamic between the interviewer and the interviewee. Our findings show that using a mobile phone further expands the freedom for the participants, and inevitably means a redistribution of power from the researcher to the participants. At the same time, the interviewer controls most elements in the interview, such as the topics discussed [53]. The redistribution of power can, therefore, be both welcomed and challenging. 

Regarding other aspects having to do with the theme *relational and social aspects*, we also reflected on how the participants´ sense of emotional ease contrasted with the researchers’ feelings of being less able to recognize and respond to the participants´ emotions and states of mind. A lack of visible feedback meant a need to use the voice and the language more consciously to convey understanding and show interest in the participant’s unique experiences.

I can hear that she is sad. I tell her this and say something confirmatory. I emphasize that it is ok to take a break if she wants to. (Written by E.S. The quote refers to a female participant, 38 years, stress syndrome)

In an in-person interview or video-based option, it is possible to non-verbally assure participants that their stories are ‘on track’, or show sympathy and understanding, in order to not disrupt them. In a telephone interview, however, the nod of the head must be made audible, all the while avoiding interrupting the interviewee. For the interviewer, this involves a clear shift from the non-verbal feedback style to the audible.

She is crying, which she had hinted might happen the first time that we talked. I tell her that we can take a break or end the interview if needed. Not seeing the other person makes it more difficult for me to decide whether to continue or not. I must trust her. It is apparent that the verbal response becomes more important when someone is showing emotion. (Written by A.A. The quote refers to a female participant, 35 years, multimorbidity)

An advantage, however, was that the format of the telephone interview seemed to enrich the participants’ stories. For example, the participants themselves conveyed that being behind *the telephone acted as a ‘shield’*, which, in a sense, allowed them to more easily express themselves, and we reflected over the openness and details in the participants’ stories. For some, the possibility to choose their level of emotional closeness or distance meant that they were more comfortable talking about sensitive subjects.

I am surprised to see that their stories have a flow to them, that they have shared openly. They also reflect on this themselves, that the anonymity allows an openness. (Written by L.H.)

## 4. Reflections and Strategies for Conducting Telephone Interviews—Before, during and after

The results point to the importance of telephone interviews by decreasing emotional demands put on the participants, focusing the importance of anonymity and social responsibility, and providing the participants with the freedom to choose level of intimacy, but also contributing to research despite dealing with symptoms. Although the ongoing COVID-19 pandemic obliged us, as researchers, to conduct interviews by phone, some participants regarded the mobile phone option as a crucial factor which enabled them to participate in a research interview. These results are important to address in future studies, because the participants—often struggling with symptoms such as pain, exhaustion, or anxiety—had to spend less energy on paying attention to social cues and norms, and could instead focus on how to reveal their personal experiences. 

More information about the informal insights derived from qualitative interviews as a means for data has been called for [33]. Our findings highlight challenges, advantages, and possible strategies which can be useful (1) when preparing the interview, (2) during the interview, and (3) after the interview. These strategies are relevant for all telephone interviews with participants where some are particularly important for the study group, i.e., participants struggling with symptoms such as pain, exhaustion, or anxiety.

When preparing the interview, our findings indicate the importance of a first introductory call to familiarize the interviewer and interviewee with each other and discuss how the interview will be carried out. This entails telling participants that they should preferably be able to talk freely without distraction, and that silence during the interview should be interpreted as active listening from an interviewer who does not want to disturb their stories. This introductory conversation is to prepare the participant for the particular form of dialog that a telephone interview is, but it also serves to establish rapport. In other words, it is a way of ‘getting to know each other’ without seeing each other, rather than clarifying the use of the voice as well as silences. This is important for building trust between the interviewee and the interviewer in line with the recommendation of, for example, Drabble et al. [32]. We also found that it was important for the interviewer to convey to the participant his or her understanding of the circumstances that are central to the subject of the interview—in our case, their health status and work disability. This suggests that the potential of the method is related to the interviewer being sufficiently familiar with the research topic and the specific kind of difficulties the participants are facing. This can be an important factor for validating the participant during the interview and building a trusting relationship over the telephone, as we were not able to do so using visual cues. As all participants used mobile phones, we found it necessary to encourage participants to choose a place where they have good reception and minimal background noise, especially important when using mobile phone compared to landline phone. This can prevent problems arising during the telephone interview and allay the researcher’s own worries beforehand. The researcher too must choose a space with good reception and check that the recording equipment is working properly.

During the interviews, we found that verbalization was important for communicating the reason for silences (e.g., taking notes or giving time for the participant to continue talking). Communicating responses was also important (e.g., saying ‘please continue’, ‘do you need to take a break’, or giving short summaries of what had been said). In addition, the tone of voice was found to be another important tool for conveying interest and understanding, as well as establishing confidence. Further, we found that asking participants about their experience of being interviewed over the telephone was a good way of ending the interview, which primarily was about their experiences of being on sick leave. This smoothly closed the main story, allowed the participants to be brought back to the present and gave them the power of being experts in their own experience of the interview situation.

After the interviews, we found it important to gather our own reflections and experience of the interview by writing summaries of our overall impressions and making field notes about our experience of the interviewing situation as well as the main findings in relation to the questions asked. These field notes were valuable tools for evaluating or supplementing the data and they were used as data for the researcher’s reflections in the findings [43]. As we did not have to spend any time traveling to or from the interviews, we were able to carry out this post-interview part of the procedure more effectively, directly after the interview. Completing the interviews from home or the workplace for us as researchers also meant that we could secure the data in an effective way, i.e., we could save the recording in a secure manner immediately after the interview was over. 

## 5. Methodological Considerations

There are a lack of methodological studies which investigate the use of telephone interviews with individuals with CMD and/or multimorbidity, where this study contributes to the gap in the literature. The strategic sampling of participants, with a diversity in demographic characteristics and viewpoints, facilitates the provision of a rich data set [54]. Yet, transferability of findings from qualitative studies may be limited to other groups or settings. To allow for judgment of transferability to other groups or setting, the authors strived to provide detailed descriptions of study design and clear communication of the findings. Although some of the findings are specifically related to the participants symptoms from their CMDs and/or multimorbidity, they may also be transferable to other groups which may not have a diagnosis but do experience the same type of symptoms or difficulties.

A limitation with the study is that the participants in general did not have experience of in-person or internet-based research interviews and that we did not have a comparison group who conducted the interviews in person or via internet-based option. However, as our purpose was not to compare the different formats but rather to gather knowledge on the experience of the telephone interviews from the perspective of participants, this was also beyond our scope. One might also want to consider how the presence of a third person during the interviews could have constrained the participants’ responses; however, we do not have information about the presence of other people, besides children being present during the interviews. Furthermore, in cases where the participants were in public, we rescheduled interviews to a better suited time and setting.

## 6. Conclusions

To conclude, telephone interviews are a method with both advantages and challenges. They provide more anonymity which seem to have a positive effect on self-disclosure and emotional display, while making fewer demands of participants in terms of emotion work and social responsibility. However, the shift from nonverbal to the audible put higher demands on the use of voice and require more intense listening on both parts. Worries about the quality of the interview due to difficulties with technology and sound or disturbances in the environment are also challenges presented as well as the loss of human encounter. Using telephone interviews as a means of qualitative data collection balance the power relationship between the interviewer and the interviewee, which can be demanding for the interviewer but beneficial for those being interviewed. The advantages, which were deemed as more important than the challenges, may give a certain group of individuals (e.g., those with CMDs or multimorbidity) a fairer opportunity to participate in research projects and share their experiences. Telephone interviews can be regarded as a valuable first option if the purpose of the study is not to build a relationship over time or observe visual cues, but rather about how people experience their lives. 

## Figures and Tables

**Table 1:** Information about the overall aim of respective project and study, recruitment, and procedure.

Project	RECO-Project	PROSA-Project	
Study	I	II	III
Overall aim of study	To explore how people with multimorbidity who were on SA experienced the support of a rehabilitation coordinator during the rehabilitation and RTW process.	To explore employees’ experience of taking part in the intervention and ethical issues that arise from the intervention.	To analyze the employees’ and employers’ experience of causes of sick leave due to CMD, barriers, and facilitating factors in private and working life for RTW.
Diagnoses	Multimorbidity (e.g., CMD, neuropsychiatric disorder, pain disorder, addiction, and other somatic diagnoses). These diagnoses are based on self-reports.	CMD These diagnoses are based on the main diagnoses on the sick leave medical certificate.	CMD These diagnoses are based on the main diagnoses on the sick leave medical certificate.
Recruitment and contact	First contact with rehabilitation coordinators delivering the intervention.	First contact with research assistant.	First contact with researcher.

RECO = The rehabilitation coordinator project; PROSA = A problem solving intervention in primary health care aimed at reducing sick leave among people suffering from common mental disorders – a cluster-randomized trial; SA = sickness absence; CMD = common mental disorders; RTW = return to work; I, II, III = refers to the three different projects in which data was collected from.

**Table 2:** Sociodemographic characteristics of the participants (*n* = 32).

Characteristics	*n* = 32
Gender	
Female	25
Male	7
Age	
Mean years (range)	44.1 (22–62)
Sick leave	
Full-time	8
Part-time	10
Returned to work/in school	14
Occupation	
Office work	17
Manual	12
In school	1
Unemployed	2

## Data Availability

The data presented in this study are available on request from the authors V.V. and E.B.B. The data are not publicly available due to ethical restrictions.

## References

[1] Denzin N.K., Lincoln Y.S. (2011). The SAGE Handbook of Qualitative Research.

[2] Kvale S., Brinkmann S. (2009). Interviews: Learning the Craft of Qualitative Research Interviewing.

[3] Rubin H.J., Rubin I.S. (2011). Qualitative Interviewing: The Art of Hearing Data.

[4] Adhabi E., Anozie C.B. (2017). Literature review for the type of interview in qualitative research. Int. J. Educ..

[5] DiCicco-Bloom B., Crabtree B.F. (2006). The qualitative research interview. Med. Educ..

[6] McGrath C., Palmgren P.J., Liljedahl M. (2019). Twelve tips for conducting qualitative research interviews. Med. Teach..

[7] Turner D.W. (2010). Qualitative interview design: A practical guide for novice investigators. Qual. Rep..

[8] Ogden T., Fixsen D.L. (2014). Implementation Science: A Brief Overview and a Look Ahead. J. Psychol..

[9] McCoyd J.L., Kerson T.S. (2006). Conducting intensive interviews using email: A serendipitous comparative opportunity. Qual. Soc. Work.

[10] Lobe B., Morgan D., Hoffman K.A. (2020). Qualitative data collection in an era of social distancing. Int. J. Qual. Methods.

[11] Sy M., O’Leary N., Nagraj S., El-Awaisi A., O’Carroll V., Xyrichis A. (2020). Doing interprofessional research in the COVID-19 era: A discussion paper. J. Interprof. Care.

[12] Teti M., Schatz E., Liebenberg L. (2020). Methods in the Time of COVID-19: The Vital Role of Qualitative Inquiries. Int. J. Qual. Methods.

[13] Nind M., Coverdale A., Meckin R. (2021). Changing Social Research Practices in the Context of Covid-19: Rapid Evidence Review. National Centre for Research Methods. Economic and Social Research Council.

[14] Johnson D.R., Scheitle C.P., Ecklund E.H. (2019). Beyond the In-Person Interview? How Interview Quality Varies Across In-person, Telephone, and Skype Interviews. Soc. Sci. Comput. Rev..

[15] Irvine A., Drew P., Sainsbury R. (2013). Am I not answering your questions properly? Clarification, adequacy and responsiveness in semi-structured telephone and face-to-face interviews. Qual. Res..

[16] Jones H., Cipriani A. (2019). Barriers and incentives to recruitment in mental health clinical trials. Évid. Based Ment. Health.

[17] Treweek S., Pitkethly M., Cook J., Fraser C., Mitchell E., Sullivan F., Jackson C., Taskila T.K., Gardner H. (2018). Strategies to improve recruitment to randomised trials. Cochrane Database Syst. Rev..

[18] Patel M.X., Doku V., Tennakoon L. (2003). Challenges in recruitment of research participants. Adv. Psychiatr. Treat..

[19] Hermanowicz J.C. (2002). The great interview: 25 strategies for studying people in bed. Qual. Sociol..

[20] Novick G. (2008). Is there a bias against telephone interviews in qualitative research?. Res. Nurs. Health.

[21] Glogowska M., Young P., Lockyer L. (2011). Propriety, process and purpose: Considerations of the use of the telephone interview method in an educational research study. High. Educ..

[22] Shuy R.W., Gubrium J.F., Holstein J.A. (2002). In-person versus telephone interviewing. Handbook of Interview Research: Context and Method.

[23] Ward K., Gott M., Hoare K. (2015). Participants’ views of telephone interviews within a grounded theory study. J. Adv. Nurs..

[24] Cachia M., Millward L.J. (2011). The telephone medium and semi-structured interviews: A complementary fit. Qual. Res. Organ. Manag. Int. J..

[25] Holt A. (2010). Using the telephone for narrative interviewing: A research note. Qual. Res..

[26] Sturges J., Hanrahan K. (2004). Comparing telephone and face to face qualitative interviewing: A research note. Qual. Res..

[27] Vogl S. (2013). Telephone versus face-to-face interviews: Mode effect on semistructured interviews with children. Sociol. Methodol..

[28] Krouwel M., Jolly K., Greenfield S. (2019). Comparing Skype (video calling) and in-person qualitative interview modes in a study of people with irritable bowel syndrome–an exploratory comparative analysis. BMC Med. Res. Methodol..

[29] Irvine A. (2011). Duration, dominance and depth in telephone and face-to-face interviews: A comparative exploration. Int. J. Qual. Methods.

[30] Trier-Bieniek A. (2012). Framing the telephone interview as a participant-centred tool for qualitative research: A methodological discussion. Qual. Res..

[31] Carr E.C., Worth A. (2001). The use of the telephone interview for research. Nurs. Times Res..

[32] Drabble L., Trocki K.F., Salcedo B., Walker P.C., Korcha R.A. (2016). Conducting qualitative interviews by telephone: Lessons learned from a study of alcohol use among sexual minority and heterosexual women. Qual. Soc. Work.

[33] Farooq M.B., De Villiers C. (2017). Telephonic qualitative research interviews: When to consider them and how to do them. Meditari Account. Res..

[34] King N., Horrocks C., Horrocks C. (2010). Remote interviewing. Interviews in Qualitative Research.

[35] Block E.S., Erskine L. (2012). Interviewing by telephone: Specific considerations, opportunities, and challenges. Int. J. Qual. Methods.

[36] Kee K.F., Schrock A.R. (2020). Telephone Interviewing as a Qualitative Methodology for Researching Cyberinfrastructure and Virtual Organizations. Second Int. Handb. Internet Res..

[37] Beaver K., Williamson S., Chalmers K. (2010). Telephone follow-up after treatment for breast cancer: Views and experiences of patients and specialist breast care nurses. J. Clin. Nurs..

[38] Newman D., O’Reilly P., Lee S.H., Kennedy C. (2017). Challenges in accessing and interviewing participants with severe mental illness. Nurse Res..

[39] Internetstiftelsen Svenskarna och Internet (Swedes and the Internet) 2020, 181. https://svenskarnaochinternet.se/rapporter/svenskarna-och-internet-2020/.

[40] Svärd V., Friberg E., Azad A. (2021). How people with multimorbidity and psychosocial difficulties experience support by rehabilitation coordinators during sickness absence. J. Multidiscip. Healthc..

[41] Azad A., Svärd V. (2021). Patients’ with Multimorbidity and Psychosocial Difficulties and Their Views on Important Professional Competence for Rehabilitation Coordinators in the Return-to-Work Process. Int. J. Environ. Res. Public Health.

[42] Brämberg E.B., Holmgren K., Bültmann U., Gyllensten H., Hagberg J., Sandman L., Bergström G. (2018). Increasing return-to-work among people on sick leave due to common mental disorders: Design of a cluster-randomized controlled trial of a problem-solving intervention versus care-as-usual conducted in the Swedish primary health care system (PROSA). BMC Public Health.

[43] Geertz C. (1973). The Interpretation of Cultures.

[44] Braun V., Clarke V. (2006). Using thematic analysis in psychology. Qual. Res. Psychol..

[45] Clarke V., Braun V. (2018). Using thematic analysis in counselling and psychotherapy research: A critical reflection. Couns. Psychother. Res..

[46] Nelson J. (2016). Using conceptual depth criteria: Addressing the challenge of reaching saturation in qualitative research. Qual. Res..

[47] Atkinson P. (1992). The ethnography of a medical setting: Reading, writing, and rhetoric. Qual. Health Res..

[48] Seitz S. (2016). Pixilated partnerships, overcoming obstacles in qualitative interviews via Skype: A research note. Qual. Res..

[49] Sipes J.B., Roberts L.D., Mullan B. (2019). Voice-only Skype for use in researching sensitive topics: A research note. Qual. Res. Psychol..

[50] Whale K. (2017). The use of Skype and telephone interviews in sensitive qualitative research with young people: Experiences from the ROCCA continence study. Qual. Methods Psychol. Bull..

[51] Hochschild A. (1979). Emotion work, feeling rules and social structure. Am. J. Sociol..

[52] Goffman E. (1978). The Presentation of Self in Everyday Life.

[53] Brinkmann S., Kvale S. (2005). Confronting the ethics of qualitative research. J. Constr. Psychol..

[54] Williams E.N., Morrow S.L. (2009). Achieving trustworthiness in qualitative research: A pan-paradigmatic perspective. Psychother. Res..

